# A possible dose–response association between distance to farmers’ markets and roadside produce stands, frequency of shopping, fruit and vegetable consumption, and body mass index among customers in the Southern United States

**DOI:** 10.1186/s12889-016-3943-7

**Published:** 2017-01-11

**Authors:** Stephanie B. Jilcott Pitts, Jedediah Hinkley, Qiang Wu, Jared T. McGuirt, Mary Jane Lyonnais, Ann P. Rafferty, Olivia R. Whitt, Nancy Winterbauer, Lisa Phillips

**Affiliations:** 1 Department of Public Health, Brody School of Medicine East Carolina University, 600 Moye Blvd, MS 660, Lakeside Annex 7, Greenville, NC 27834 USA; 2 Healthy Foods Coordinator Partnerships to Improve Community Health, Albemarle Regional Health Services, Elizabeth City, NC USA; 3 Department of Biostatistics, East Carolina University, 2435D Health Sciences Building, Greenville, NC 27834 USA; 4 Department of Nutrition, University of North Carolina at Chapel Hill, Chapel Hill, NC 27599 USA

**Keywords:** Farmers’ market, Community nutrition, Fruit, Vegetable, Obesity, Consumer behavior

## Abstract

**Background:**

The association between farmers’ market characteristics and consumer shopping habits remains unclear. Our objective was to examine associations among distance to farmers’ markets, amenities within farmers’ markets, frequency of farmers’ market shopping, fruit and vegetable consumption, and body mass index (BMI). We hypothesized that the relationship between frequency of farmers’ market shopping and BMI would be mediated by fruit and vegetable consumption.

**Methods:**

In 15 farmers’ markets in northeastern North Carolina, July–September 2015, we conducted a cross-sectional survey among 263 farmers’ market customers (199 provided complete address data) and conducted farmers’ market audits. To participate, customers had to be over 18 years of age, and English speaking. Dependent variables included farmers’ market shopping frequency, fruit and vegetable consumption, and BMI. Analysis of variance, adjusted multinomial logistic regression, Poisson regression, and linear regression models, adjusted for age, race, sex, and education, were used to examine associations between distance to farmers’ markets, amenities within farmers’ markets, frequency of farmers’ market shopping, fruit and vegetable consumption, and BMI.

**Results:**

Those who reported shopping at farmers’ markets a few times per year or less reported consuming 4.4 (standard deviation = 1.7) daily servings of fruits and vegetables, and those who reported shopping 2 or more times per week reported consuming 5.5 (2.2) daily servings. There was no association between farmers’ market amenities, and shopping frequency or fruit and vegetable consumption. Those who shopped 2 or more times per week had a statistically significantly lower BMI than those who shopped less frequently. There was no evidence of mediation of the relationship between frequency of shopping and BMI by fruit and vegetable consumption.

**Conclusions:**

More work should be done to understand factors within farmers’ markets that encourage fruit and vegetable purchases.

## Background

In the United States, there is greater obesity among rural versus urban populations [1, 2]. Factors in both the community and consumer food environments are associated with dietary behaviors and subsequent obesity [3]. The community food environment includes community- or neighborhood-level access to healthier foods via retail food outlets (e.g., supermarkets, farmers’ markets) [3]. The consumer food environment includes characteristics within retail food outlets that either promote or hinder healthier food and beverage purchase (e.g., healthier foods placed in check-out aisles) [3]. Adding new farmers’ markets is one strategy to increase access to healthy foods in rural areas, improving both the community and consumer food environments [4].

There are associations between the consumer and community food environment and purchase and consumption of healthier foods: Individuals who live closer to chain supermarkets and farmers’ markets (community food environment) have healthier diets and lower body mass index (BMI) [5–7]. Furthermore, prior studies have found that shopping at farmers’ markets is associated with greater self-reported fruit and vegetable consumption [8–10]. In chain supermarkets and other large food stores, there is some evidence that price promotions, and other marketing strategies (consumer food environment) are associated with healthier purchases [11–14]. Thus, in a similar way, healthy food access might be bolstered not only by creating new farmers’ markets, but also by providing an improved consumer food environment within the farmers’ market [15]. Focusing on the community and consumer food environments aligns with the 5 dimensions of food access proposed by Caspi and colleagues, [16] based off of Penchansky and Thomas’s model of health care access, [17] including availability (i.e., adequacy of healthy foods), accessibility (travel time and distance to food retail outlets), affordability (food prices), accommodation (attitudes about the food environment), acceptability (how well local food sources adapt to residents’ needs) [16].

**Fig. 1:**
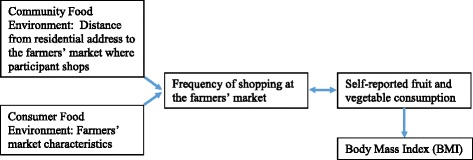
Conceptual model to examine associations between distance to farmers’ markets, farmers’ market characteristics, frequency of farmers’ market shopping, self-reported fruit and vegetable consumption, and body mass index (BMI)

The prevailing hypothesis of these and similar studies is that people shop at markets closest to home and may also shop more frequently and buy more fresh fruits and vegetables (perishable goods) when a market is closer to the residential address. However, studies are finding that individuals do not shop at supermarkets or farmers’ markets closest to home [9, 18, 19]. It could be that elements of the consumer food environment are what motivate individuals’ shopping behaviors, more than just distance alone. Thus, we examined cross-sectional associations between distance to farmers’ markets and roadside produce stands, frequency of farmers’ market and produce stand shopping, fruit and vegetable consumption, and BMI, testing the prevailing hypothesis that those who live closer to farmers’ markets and stands will shop more frequently at those markets and stands (versus those who live further from markets and stands, who will shop less frequently), and also consume more fruits and vegetables and have a lower BMI. We also hypothesized that the relationship between frequency of farmers’ market shopping and BMI would be mediated by fruit and vegetable consumption. Furthermore, we examined associations between farmers’ market characteristics (e.g., signage, payment options, availability of fruits and vegetables), frequency of farmers’ market shopping, and fruit and vegetable consumption among 263 customers in 15 farmers’ markets and roadside produce stands in northeastern North Carolina. The conceptual model undergirding these analyses is in Fig. 1.

## Methods

### Study setting and participants

This cross-sectional study took place in a 17 county region in northeastern North Carolina, as part of the evaluation of the Centers for Disease Control and Prevention (CDC)-funded Albemarle Regional Health Services (ARHS) Partnerships to Improve Community Health (PICH) grant. The 17-county PICH region consists of mostly non-metro, rural counties with higher rates of poverty and obesity than the rest of the state [20, 21]. This region is primarily agricultural and sparsely populated. For example, one of the PICH counties, Gates County, has a 2015 population estimate of 11,431 persons, and a 2010 population per square mile of 35.8. Bertie County has a population of 20,199 persons, and a population density of 30.4. A few of the more highly populated PICH counties have populations and population densities of 39,829 and 179.2 persons per square mile (Pasquotank County), and 54,150 and 111.9 persons per square mile (Edgecombe County) [21]. These counties have high rates of adult obesity (ranging from 29.2% for Currituck to 37.0% for Edgecombe), and often have very few retail outlets offering healthy food and beverage options, highlighting the importance of direct farm-to-consumer outlets in the PICH regions.

Trained surveyors recruited a convenience sample of farmers’ market customers between July 2015 and September 2015 at farmers’ markets (*n* = 7) and roadside produce stands (*n* = 8) located in 14 of the 17 county region of northeastern North Carolina. (For the purposes of this paper, markets and stands are referred to as “farmers’ markets”.) Between 5 and 46 customers were surveyed at each market. Eligibility criteria were being English-speaking and over 18 years of age. Potential participants were approached at the entrance of the farmers’ market and asked if they would be interested in participating in a survey about farmers’ markets. If the participant agreed (verbal consent), (s)he was given a 5-page questionnaire, and if requested, the questions were read aloud. This study was reviewed and approved by the East Carolina University Institutional Review Board (15–000427). Study participants were given a reusable grocery tote upon questionnaire completion.

### Frequency of farmers’ market shopping and barriers to and motivators of farmers’ market shopping

Participants were queried about their frequency of farmers’ market shopping by asking “How often in the past 12 months did you buy fruits or vegetables locally grown from a farmer’s market, CSA (community supported agriculture), roadside stand, or pick-your-own produce farm?” Response options included 2 or more times per week, one time per week, 2–3 times per month, once a month, a few times per year, and never. Due to distribution of responses, there were four categories established: A few times per year or less (combination of a few times per year and never); One to several times per month (combination of once a month and 2–3 times per month); Once a week; and 2 or more times per week.

Participants could select from a list of motivators of and barriers to shopping at farmers’ markets, used in a prior study, [8] with motivators including support local farmers, fresher produce, better prices, and variety of the products, and barriers including no Supplemental Nutrition Assistance Program (SNAP), Electronic Benefit Transfer (EBT), no credit/debit accepted, market days and hours aren’t convenient, and “I get what I need from other places.” Participants could also select “other” and write in responses. The perceived relative expense of produce at farmers’ markets versus supermarkets was assessed by asking “compared to other places you purchase fruits and vegetables, is the farmers’ market more or less expensive?” with response options including more expensive, less expensive, the same price, and it depends.

### Fruit and vegetable purchase and consumption and body mass index

The questionnaire also assessed whether fruit and vegetable purchase and consumption had increased given shopping at farmers’ markets using the following question: “As a result of your shopping at this Farmers’ Market, have you been eating more fruits and/or vegetables than before you started to shop here?” Response options were fewer fruits/vegetables, no change, more fruits and vegetables, and “this is my first time at this market.” The questionnaire also assessed the proportion of fruits/vegetables purchased at farmers’ markets relative to other goods. The number of servings of fruits and vegetables eaten per day was assessed using the following items: “On a typical day, how many servings of fruits do you eat? (A serving of fruit is like a medium sized apple or a half cup of fresh fruit. – this does not include fruit juice)” and “On a typical day, how many servings of vegetables do you eat, not including French fries? (A serving of vegetables is like one cup of green salad or half a cup of cooked vegetables.)” Response options ranged from 1 to 6 or more servings per day, and this was summed as self-reported fruit and vegetable consumption. These questions were similar to the “Food Within Reach” assessment and the items on the Child Health Assessment and Monitoring Program (CHAMP) [22, 23]. Body mass index was calculated from self-reported height and weight, as weight in kilograms divided by height in meters squared. BMI was corrected for systematic reporting error for height and weight using a method previously described [24].

### Demographic information

We also collected customers’ residential addresses for Geographic Information Systems Analyses. Customer demographics included assessment of age (in years), sex, ethnicity, race, income, and educational attainment.

### Farmers’ market amenities (Consumer food environment)

Farmers’ market audits were conducted to quantify amenities offered at each farmers’ market (e.g., payment methods, farmers’ market signage, fruits and vegetables available). The audits were completed by trained auditors (who were also administering the customer intercept questionnaires) on the day the intercept surveys were conducted. The audit included items from the validated Farmers’ Market Audit Tool (FMAT) [25] and the North Carolina Fruit and Vegetable Outlet Inventory (NCFVOI) Tool [26]. Audit data were entered into a Qualtrics survey by the auditor. To quantify the amenities offered at each market, a farmers’ market amenities index was created based upon the farmers’ market audit data. The index consisted of whether or not SNAP/EBT was accepted (0 = no, 1 = yes), the forms of payment accepted (1 point for each form of payment accepted, of cash, check, credit/debit, SNAP, WIC), farmers’ market sign (yes/no), sign promoting SNAP/EBT, a welcome booth, and availability of 17 types of fruits and vegetables (coded as the number of vendors selling that item). The amenities index was a sum of all characteristics, and ranged from 5 to 38. We also created a fruit and vegetable availability sub-score, and used this in analyses. The score was a sum of the number of vendors selling each of 17 fruit and vegetable items, and ranged from 4 to 28.

### Geographic information systems (GIS) mapping (Community food environment)

To learn more about the shopping patterns of farmers’ market shoppers, a GIS database was created. For the GIS analyses, all respondents who did not provide their address or did not live in the 17-county PICH region were omitted from the GIS analyses. Of the 263 who were surveyed, 31 lived outside a PICH county, and 33 did not give an address that was complete enough for geocoding, leaving 199 respondents for GIS analyses. All addresses were geocoded to the highest level of accuracy possible, either to the city centroid, street centroid, or to the rooftop level. Addresses of all farmers’ markets in the 17 county area and all surveyed customers were batch geocoded using the Google Maps geocoding Application Programming Interface (API) through the BatchGeo website. Address data were verified using Google maps and satellite images. Distances from participant’s home address to the closest farmers’ market, as well as the distance from their home to the farmers’ market where they completed the survey (if different from the closest farmers’ market), were calculated using ArcGIS Spatial Analyst. Distances were calculated over an integrated statewide street network to reduce edge effects and to account for customers’ ability to traverse county boundaries. Three GIS variables were calculated: (1) The distance (in miles) to the closest farmers’ market from the participant’s residential address; (2) the distance (in miles) to the farmers’ market where the participant was surveyed; and (3) the difference between the two distances, which would be zero if the participant was surveyed at the farmers’ market closest to his or her residential address.

### Statistical analyses

Customer and market characteristics were analyzed using descriptive statistics, including means and standard deviation for continuous variables, and frequencies for categorical variables. Bivariate associations included correlation (two continuous variables), t-tests and Analysis of variance (for a categorical and a continuous variable), and for two categorical variables, a chi-square analysis of independence. To examine potential differences between farmers’ markets and roadside produce stands, we used t-tests and Fisher’s exact tests to examine the differences between farmers’ markets and produce stands in terms of overall amenities score, fruit and vegetable availability, sign availability, and SNAP/EBT availability.

To examine associations hypothesized in our conceptual model in Fig. 1, we used multinomial logistic regression analyses (adjusted for age, race, sex, and education) to examine associations between frequency of farmers’ market shopping (dependent variable) and distance to farmers’ market and amenities index for markets (both used as independent variables in separate analyses). The multinomial logistic regression used “a few times per year or less” as the reference group. Poisson and linear regression were used to examine the association between farmers’ market shopping frequency (independent variable) and separate dependent variables of (1) fruit and vegetable consumption and (2) BMI, respectively, adjusting for age, race, sex, and education. Poisson regression analyses were also used to examine associations between fruit and vegetable consumption and the farmers’ market amenities index. We examined the need for multi-level models, with farmers’ markets as the second level, but the random effects were not statistically significant, suggesting there was no need for multi-level models. We examined potential mediation of the relationship between shopping frequency and BMI by fruit and vegetable consumption using Baron and Kenny criteria [27]. This was an exploratory study, and we did not conduct an a priori power analysis. All analyses were conducted in SAS version 9.4 (SAS Institutes, Cary, North Carolina).

### Availability of data and materials

The datasets generated and analyzed for this project are not publicly available due to participant confidentiality, but de-identified datasets may be available from the corresponding author on reasonable request.

## Results

Characteristics of farmers’ market shoppers are provided in Table 1. Customers (total *n* = 263) had a mean age of 56 years, mean BMI of 29 kg/m^2^, reported consuming a mean of 5 servings of fruits and vegetables daily, and a large majority had an income of over $40,000 per year. The main motivators to shopping at farmer’s markets were fresher produce, support for local farmers, produce tastes better, and friendly atmosphere. The main barriers to shopping at farmers’ markets were market days/h are not convenient, out of the way, and “I get what I need from other places.” Thirty seven percent (37%) of customers shopped at the farmers’ market or produce stand closest to their residential address (data not shown), 60% said they had increased fruit and vegetable consumption as a result of shopping at farmers’ markets and 49% said they had increased the variety of fruits and vegetables consumed as a result of farmers’ market shopping. (Table 1) Over 65% of respondents purchased a majority (75% or more) of fruits and vegetables at markets. The mean distance to the closest farmers’ market was 3.9 miles, whereas the mean distance to the market where the individual was surveyed was 7.0 miles. We examined differences between geocoded and non-geocoded participants. The non-geocoded participants were more educated (64% vs. 49% college graduates) and younger (50.3 vs 57.0 years mean age) compared to the geocoded participants.

**Table 1:** Participant characteristics for 263 farmers’ market customers surveyed at 15 different farmers markets and roadside produce stands in northeastern North Carolina

Characteristic	N	Mean	Standard Deviation
Age (years)	251	55.6	16.5
Body mass index (kg/m^2^)	219	28.7	7.0
Fruit (servings/day)	253	2.5	1.3
Vegetables (servings/day)	254	2.6	1.2
Fruits and vegetables (servings/day)	252	5.0	2.1
Typical amount spent on produce at a farmers’ market (dollars)	245	18.9	14.7
Geographic Information System (GIS) measured distance to closest farmers’ market from residential address (miles)	196	3.9	3.9
GIS measured distance from the residential address to the farmers’ market where participant was surveyed (miles)	196	10.9	18.4
Difference in GIS measured distance between the market where the participant was surveyed and the market closest to home (miles)	196	7.0	17.7
Characteristic	N	Frequency	Percentage
Gender (% female)	255	186	72.9
Education (% with some college or more)	252	192	76.2
Race (% black)	248	50	20.2
Race (% white)	248	181	73.0
Race (% other)	248	17	6.9
Ethnicity (% Hispanic)	227	6	2.6
Income (% over 40,000)	185	114	61.6
Currently receive WIC (% yes)	252	11	4.4
Redeemed WIC at a farmers’ market (% yes)	263	7	2.7
Currently receive SNAP (% yes)	251	18	7.2
Used SNAP at Farmers’ Market (% yes)	263	3	1.1
Participate in Senior Farmers’ Market Nutrition Program (% yes)	263	5	1.9
Servings of fruit	253	Frequency	Percentage
1 per day		63	24.9
2 per day		82	32.4
3 per day		59	23.3
4 per day		31	12.3
5 per day		10	4.0
6 or more per day		8	3.2
Servings of vegetables	254	Frequency	Percentage
1 per day		33	13.0
2 per day		121	47.6
3 per day		49	19.3
4 per day		34	13.4
5 per day		10	3.9
6 or more per day		7	2.8
Self-reported increase in fruit and vegetable consumption as a result of shopping at farmers’ markets (% yes)	253	151	59.7
Self-reported increase in variety of fruit and vegetable consumed as a result of shopping at farmers’ markets (% yes)	252	123	48.8
Frequency of shopping at a farmers’ market	257	Frequency	Percentage
A few times per year		40	15.6
2–3 times per month		28	10.9
Once per month		25	9.7
One time per week		104	40.5
2 or more times per week		49	19.1
Proportion of produce purchased at farmers’ markets compared to other goods	254	Frequency	Percentage
0–24% produce		26	10.2
25–49% produce		23	9.1
50–74% produce		36	14.2
75–99% produce		89	35.0
100% produce		80	31.5
Fruits and vegetables are less expensive at the farmers’ market compared to other places (n, % yes)	252	125	49.6
Motivators for shopping at farmers’ markets (n, % yes)	263	Frequency	Percentage
Support local farmers		103	39.2
Fresher Produce		123	46.8
Produce tastes better		63	24.0
Better prices		23	8.8
It is close to home		32	12.2
It is close to work		3	1.1
Produce is grown with fewer pesticides		33	12.6
Good service		40	15.2
Quality of products		58	22.1
Variety of products		27	10.3
Consistency of the products		13	4.9
Convenient Location		32	12.2
Friendly atmosphere		63	24.0
Barriers to shopping at farmers’ markets	263	Frequency	Percentage
No Supplemental Nutrition Assistance Program		9	3.4
No credit or debit accepted		22	8.4
Not enough money to shop		11	4.2
No transportation to market		2	0.8
Prices are too high		10	3.8
Extreme weather		13	4.9
Not enough parking		3	1.1
Market days and hours aren’t convenient		46	17.5
Out of the way		37	14.1
I get what I need from other places		24	9.1
Do not know where markets are		11	4.2

Characteristics of farmers’ markets (*n* = 7) and roadside produce stands (*n* = 8) are in Table 2. Three out of the 15 markets and stands accepted SNAP/EBT. A large majority of markets had a sign and welcome booth. As seen in Table 2, while farmers’ markets tended to have higher mean amenities scores, fruit and vegetable availability, and were more likely to accept SNAP/EBT, markets and stands were not statistically different on any of these factors.

**Table 2:** Characteristics and comparison of farmers’ markets (*n* = 7) and roadside produce stands (*n* = 8) in northeastern North Carolina

Number and percentage of farmers’ markets with the following characteristics	Number	Percentage
Forms of Payment		
Accepts cash	15	100
Accepts credit/debit	7	47
Accepts Check	11	73
Accepts WIC	0	0
Accepts SNAP/EBT	3	20
Has a farmers’ market sign	13	87
Has a welcome booth	9	60
Number and percentage with the following fruits and vegetables (Audits conducted July–September 2015)	Number	Percentage
Apples	11	73
Blueberries	8	53
Cantaloupe	11	73
Peaches	14	93
Strawberries	3	20
Broccoli	2	13
Cabbage	6	40
Cauliflower	0	0
Corn	9	60
Cucumbers	13	87
Kale	5	33
Lettuce	5	33
Onions	12	80
Peppers	11	73
Squash	15	100
Tomatoes	14	93
Watermelon	13	87
Comparison of farmers’ markets and roadside produce stands	Mean	*P*-value
Total amenities score		0.2766
Farmers’ markets	20.1	
Produce stands	14.5	
Fruit and vegetable availability		0.2827
Farmers’ markets	15.4	
Produce stands	10.6	
	Percentage	*P*-value
Farmers’ market or produce stand sign		1.0000
Farmers’ markets	85.7	
Produce stands	87.5	
SNAP/EBT available		0.0769
Farmers’ markets	42.8	
Produce stands	0.0	

Using ANOVA, there was a significant bivariate association between distance to farmers’ markets and frequency of farmers’ market shopping (*P* = .049). For those who shopped “A few times per year or less”, the average distance to the market where they shopped was 17.9 miles, whereas the distances to the market for the more frequent shoppers ranged from 8 to 11 miles. (Table 3) There was a significant relationship between frequency of farmers’ market shopping and fruit and vegetable consumption (*P* = .005), such that those who shopped the least frequently also reported the fewest servings of fruits and vegetables consumed (4.4 versus 5.5 servings reported among the most frequent shoppers).

**Table 3:** Unadjusted means of distance to farmers’ market or roadside produce stand in miles, and mean fruit and vegetable consumption by shopping frequency

Frequency of farmers’ market shopping	Distance (in miles) from respondent’s home to farmers’ market or roadside produce stand where surveyed		
	*n*	Mean	Standard Deviation
A few times per year or less	34	17.87	26.05
One to several times per month	39	9.13	11.02
Once a week	83	7.97	15.71
2 or more times per week	42	10.63	17.23
Frequency of farmers’ market shopping	Servings of Fruit and Vegetable Consumed per Day		
	*n*	Mean	Standard Deviation
A few times per year or less	49	4.45	1.72
One to several times per month	53	4.47	1.62
Once a week	99	5.34	2.32
2 or more times per week	49	5.53	2.20
Frequency of farmers’ market shopping	Servings of Fruit and Vegetable Consumed per Day (Sensitivity analysis of only those participants who were geocoded.)		
	*n*	Mean	Standard Deviation
A few times per year or less	33	4.42	1.50
One to several times per month	39	4.56	1.68
Once a week	79	5.29	2.34
2 or more times per week	42	5.55	2.32

Table 4 shows regression estimates from the adjusted logistic, Poisson, and linear regression models examining associations between distance to farmers’ markets and roadside produce stands, market and stand amenities, frequency of shopping, fruit and vegetable consumption, and BMI among farmers’ market and roadside produce stand customers. In adjusted multinomial logistic regression analyses, there was a non-significant association between frequency of farmers’ market shopping and distance to farmers’ markets (*P* = .179). In adjusted Poisson regression models, fruit and vegetable consumption was significantly associated with frequency of farmers’ market shopping (*P* = .017), such that those who reported shopping at farmers’ markets more frequently consumed more fruits and vegetables than those shopping less frequently. Table 4 also indicates that the overall relationship between BMI and frequency of shopping was not statistically significant (*P* = .207), but there was a significant inverse effect for those who shopped 2 or more times per week (*P* = .034), indicating that frequent shoppers had a lower BMI. Modeling results showed no evidence of mediation of the relationship between BMI and frequency of shopping by fruit and vegetable consumption (Table 4, Row 18). There was no association between farmers’ market amenities index, the sub-score for fruit and vegetable availability, and frequency of farmers’ market shopping or fruit and vegetable consumption in bivariate analyses or adjusted models.

**Table 4:** Regression estimates from the multinomial logistic regression, Poisson regression, and linear regression models examining associations between distance to farmers’ markets and roadside produce stands, market amenities, frequency of shopping, fruit and vegetable consumption, and BMI among farmers’ market and roadside produce stand customers in northeastern North Carolina

Dependent variable	Independent variable	Parameter estimate	Standard error	*P*-value
Frequency of shopping	Distance to market or stand where surveyed			0.1796
2+ per week	Distance to market or stand where surveyed	−0.0132	0.0114	0.2459
Once a week	Distance to market or stand where surveyed	−0.0275	0.0135	0.0409
1–3 times per month	Distance to market or stand where surveyed	−0.0182	0.0136	0.1812
Frequency of shopping	Farmers’ market amenities			0.9179
2+ per week	Farmers’ market amenities	−0.0106	0.0242	0.6599
Once a week	Farmers’ market amenities	0.00425	0.0198	0.8305
1–3 times per month	Farmers’ market amenities	0.000340	0.0226	0.9880
Frequency of shopping	Farmers’ market amenities—Fruit and vegetable availability sub-score			0.8705
2+ per week	Farmers’ market amenities—Fruit and vegetable availability sub-score	−0.0193	0.0293	0.5098
Once a week	Farmers’ market amenities—Fruit and vegetable availability sub-score	0.00191	0.0234	0.9349
1–3 times per month	Farmers’ market amenities—Fruit and vegetable availability sub-score	−0.00379	0.0267	0.8873
Fruit and vegetable consumption	Frequency of shopping			0.0166
	A few times per year or less vs. 2 or more times per week	−0.1913	0.0936	0.0409
	One to several times per month vs. 2 or more times per week	−0.2330	0.0908	0.0103
	Once per week vs. 2 or more times per week	−0.0403	0.0769	0.6005
Fruit and vegetable consumption	Farmers’ market amenities	0.0034	0.0032	0.2771
Fruit and vegetable consumption	Farmers’ market amenities—Fruit and vegetable availability sub-score	0.0048	0.0037	0.1964
Body mass index	Frequency of shopping	*F* = 1.53		0.2074
	2 or more times per week vs. One to several times per month	−3.6514	1.7544	0.0388
	Once per week vs. One to several times per month	−2.3695	1.4764	0.1103
	A few times per year or less vs. One to several times per month	−2.3432	1.7032	0.1706
Body mass index	Fruit and vegetable consumption	−0.1341	0.2554	0.6002
Body mass index	Frequency of shopping	*F* = 1.44		0.2322
	2 or more times per week vs. One to several times per month	−3.6308	1.7838	0.0433
	Once per week vs. One to several times per month	−2.2390	1.5242	0.1436
	A few times per year or less vs. One to several times per month	−2.3299	1.7194	0.1771
	Fruit and vegetable consumption	−0.0652	0.2619	0.8036

(Models were adjusted for age, race, gender, and education level.)

In models adjusted for age, sex, race, and education, those who did not shop at the farmers’ market closest to their residential address reported consuming more fruits and vegetables than those who did shop at the market closest to the residential address (adjusted means of 5.3 servings of fruits and vegetables per day for those who did not shop at the closest market versus 4.4 servings per day for those who did shop at the closest market, *P* = .004). There were no significant associations between distance traveled to the farmers’ market and fruit and vegetable consumption, or the difference between the closest farmers’ market and the market at which the participant was shopping and fruit and vegetable consumption. Because we found significant differences between those who were geocoded and those not geocoded, we conducted sensitivity analyses using only those geocoded, finding similar results. For example, Table 3 shows the frequency of farmers’ market shopping and fruit and vegetable consumption for the full sample, and for only those who were geocoded, and in both cases, there is increasing consumption of fruits and vegetables with increasing frequency of farmers’ market shopping.

## Discussion

In this study, we examined cross-sectional associations between distance to farmers’ markets and roadside produce stands, frequency of farmers’ market and produce stand shopping, amenities at markets and stands, fruit and vegetable consumption, and BMI among customers in northeastern North Carolina. In the current study, the mean distance to the closest farmers’ market was 3.9 miles, whereas the mean distance to the market where the individual was surveyed was 7.0 miles. Our study findings are similar to others finding that individuals do not shop at supermarkets or farmers’ markets closest to their residential address [9, 18, 19]. This may indicate that there are factors more important than distance when individuals are determining whether to shop at a farmers’ market or stand, and likely include elements of the consumer food environment, such as prices, quality of products, or friendliness of the atmosphere.

Frequently reported motivators to shopping at farmers’ markets were fresher produce, support for local farmers, better tasting produce, and friendly atmosphere. Future studies should examine how these motivators relate to the acceptability and accommodation dimensions of food access [16]. The barriers found in this sample were similar to prior study findings [8, 9, 15] and included market days, hours and location were not convenient, and that debit/credit cards were not accepted. Addressing these barriers could lead to more farmers’ market shopping among eastern NC residents.

In this study, more frequent shopping was associated with greater fruit and vegetable consumption, and lower BMI (for the most frequent of shoppers). In addition, a majority of customers said they purchase mostly fruits and vegetables at farmers’ markets, and had increased their fruit and vegetable consumption as a result of shopping at markets. These results provide further evidence that farmers’ markets are a positive element of the community food environment. Because roadside produce stands do not have as many market amenities as farmers’ markets, but may be more frequently used due to being on the route to/from work, the inclusion of roadside produce stands may have attenuated the association between distance to markets and fruit and vegetable consumption and/or BMI. We did not find evidence of mediation of the relationship between frequency of shopping and BMI by fruit and vegetable consumption. This suggests that there may be unmeasured confounding factors, such as enjoyment of cooking among those who shopped frequently and also had lower BMIs.

One limitation of the current study is that fruit and vegetable consumption, farmers’ market shopping frequency, and weight and height were self-reported, and thus could include systematic bias. The potential serving range for fruits and vegetables provided to respondents was 1–6+, which was limited and presented a basement effect that may have biased point estimates of servings per day upward. Furthermore, the survey question regarding frequency of shopping included CSAs and pick-your-own produce farms, which are quite different from farmers’ markets and produce stands. However, at last count, of the 99 fruit and vegetable outlets in the study area, 10 were pick-your-own, and the majority of these 10 were strawberry fields, which are seasonal. Also, because we surveyed customers at farmers’ markets and roadside produce stands, we assumed that these were the markets and stands where the individual mostly shopped. This assumption should be tested in future studies. While there were not statistically significant differences between markets and stands, there was a very small sample size for that analysis.

Another major limitation is that this was a cross-sectional study, and there is the potential for reverse causation; therefore, causality cannot be assumed. For example, if customers who enjoy cooking or have higher nutritional literacy are more likely to eat fruits and vegetables, they may also shop more frequently at local farmers’ markets and stands. In this case, eating fruits and vegetables causes more frequent shopping. There were many missing addresses, causing missing data for geocoding and GIS analyses. However, we conducted sensitivity analyses to account for this, finding results largely unchanged even when the sample included only those geocoded. Northeastern North Carolina has high rates of obesity and poverty, and as such our study may have limited external validity, because the customers surveyed tended to be female, college educated, middle-aged, white, with a mean BMI of 29 kg/m^2^. Finally, there are many reasons why a person might select a particular farmers’ market, other than distance to and amenities at the market.

## Conclusions

In this study, we investigated our hypotheses in a sample of farmers’ market and produce stand customers in northeastern North Carolina, while prior farmers’ market studies examined these issues in representative or convenience community samples. We found a potential dose–response relationship between distance to farmers’ markets, frequency of farmers’ market shopping and fruit and vegetable consumption, with increasing produce consumption associated with increasing frequency of farmers’ market shopping. We also examined whether elements of the consumer food environment (e.g., payment types, welcoming atmosphere, and fruits and vegetables offered) were associated with customers’ frequency of shopping and fruit and vegetable consumption, finding that these were not associated with shopping frequency or fruit and vegetable consumption. However, the food environment within farmers’ markets has not been studied extensively in the past. Ultimately, the results of our study will inform next steps for promoting farmers’ markets in rural North Carolina and beyond.

## Abbreviations

BMI: Body mass index
CDC: Centers for disease control and prevention
CSA: Community supported agriculture
EBT: Electronic benefit transfer
FMAT: Farmers’ market audit tool
NC FVOI: North Carolina fruit and vegetable outlet inventory
PICH: Partnerships to improve community health
SNAP: Supplemental nutrition assistance program
